# Vertical Ex Vivo Dermoscopy in Assessment of Malignant Skin Lesions

**DOI:** 10.3390/biomedicines12081683

**Published:** 2024-07-28

**Authors:** Mirjana Popadić, Dimitrije Brasanac, Danijela Milošev, Ana Ravić Nikolić, Slobodanka Mitrović

**Affiliations:** 1 Department of Dermatovenerology, Faculty of Medicine, University of Belgrade, Clinic of Dermatovenereology, University Clinical Centre of Serbia, 11000 Belgrade, Serbia; 2 Institute of Pathology, Faculty of Medicine, University of Belgrade, 11000 Belgrade, Serbia; 3 Department of Pathology, University Clinical Centre Kragujevac, 34000 Kragujevac, Serbia; 4 Department of Dermatovenerology, Faculty of Medical Sciences, University of Kragujevac, University Clinical Centre Kragujevac, 34000 Kragujevac, Serbia; 5 Department of Pathology, Faculty of Medical Sciences, University of Kragujevac, University Clinical Centre Kragujevac, 34000 Kragujevac, Serbia

**Keywords:** dermoscopy, ex vivo, vertical view, histopathology, malignant skin lesions

## Abstract

The role of vertical ex vivo dermoscopy relevant to clinical diagnosis has not been investigated yet. Study objectives were defining, describing, and determining the importance of the structures visible using vertical ex vivo dermoscopy in the diagnosis of malignant skin lesions, as well as determining their accuracy in the assessment of tumor margins. A prospective, descriptive study was conducted in two University centers. Digital images of completely excised skin lesions, fixed in formalin, before histopathological diagnosis were used for analysis. BCCs had the most diverse dermoscopic presentation on the vertical section, while SCCs showed a similar presentation in most cases. Vertical dermoscopy of thin melanomas was almost identical, unlike nodular melanomas. Thickness accuracy assessed by dermatologist was 0.753 for BCC, 0.810 for SCC, and 0.800 for melanomas, whereas assessment by pathologist was 0.654, 0.752, and 0.833, respectively. The accuracy of tumor width assessment was 0.819 for BCCs, 0.867 for SCCs and 1.000 for melanoma as estimated by a Dermatologist. Interobserver agreement was 0.71 for BCC, 0.799 for SCC and 0.832 for melanomas. Vertical ex vivo dermoscopy may contribute to the distinction between BCCs, SCCs, and melanomas. Moreover, regardless of the doctor’s specialty, it enables a good assessment of the tumor’s margins.

## 1. Introduction

Hundreds of years ago, dermatologists used optical devices, primarily magnifying glasses, with or without a light source, to supplement the examination of skin lesions with the naked eye [1]. Nowadays, before histopathological analysis, dermoscopy is increasingly used as an in vivo, non-invasive diagnostic method that allows an in-depth view of the skin [2]. The importance of dermoscopy in the diagnosis and distinction, primarily of pigmented skin lesions, has been widely studied and confirmed by numerous studies [3,4]. A small number of studies have gone a step further in the investigation, introducing the horizontal ex vivo dermoscopy which involves the application of horizontal dermoscopy on freshly excised tissue [5] or tissue fixed in formalin [6,7,8]. Although horizontal ex vivo dermoscopy correlates with in vivo dermoscopy [6] its negative side is reflected in poorer image quality, loss of vascular structures and significant changes in color [9]. Nevertheless, published papers have confirmed its importance for pathologists, in determining margins and directing cross-sections during tissue sampling, pointing to the field of interest for vertical section [7,8,10].

A few papers have been published about vertical ex vivo dermoscopy, which involves the application of dermoscopy on a vertical section of excised tissue, fresh or fixed. Vertical ex vivo dermoscopy clearly differentiates skin layers as well as the anatomical location of pigment [11] and is presented as a rather useful method for analyzing pigmented structures [12]. A recent article suggests the possible utility of vertical ex vivo dermoscopy for surgeons, in mapping during Mohs micrographic surgery and easy sharing of mobile photographs of marked affected boundaries, if the Mohs laboratory is not present in the surgical area [13]. However, the role of vertical ex vivo dermoscopy in providing additional information relevant to more accurate diagnostics in clinical practice has not been investigated yet. 

## 2. Materials and Methods

After approval of the institutional Ethics committee, a prospective, descriptive study in the field of vertical ex vivo dermoscopy was conducted. The experimental part of the research was performed in two University centers. The research included patients whose lesions were completely surgically removed and sent for histopathological verification in the period from 1 September to 31 December 2019. The study was postponed due to the epidemiological situation caused by COVID and continued from 1 January to 1 April 2022.

Criteria for inclusion of patients in the study were: (1) completely surgically removed skin lesion, (2) good quality dermoscopic images, and (3) clear histopathological diagnosis. Demographic characteristics of patients and clinical data were collected by reviewing histopathological referrals and outpatient patient records.

The research was performed on surgical specimens fixed in formalin before further processing according to the protocol. Clinical images of excised tissue were made first. Afterwards, the contact surface of the dermatoscope was covered with a thin cellophane tightened with a rubber band for jars, to prevent cross-contamination [14]. By applying contact horizontal dermoscopy, the field of interest for the vertical cross-section of the tissue was determined. After dermoscopic photographing of the horizontal aspect of the skin tissue, a vertical section was made, which was also photographed according to the same, previously described, protocol.

For digital, clinical and dermoscopic images, a cell phone camera previously connected to a hand-held dermatoscope (Dermlite 3DLN, San Juan Capistrano, CA, USA) was used. Dermoscopic pictures of the vertical section were taken at different zoom magnifications. All images were electronically transferred and stored on a computer for further evaluation. For dermoscopic analysis of horizontal ex vivo dermoscopy, the pattern analysis was applied [4]. For the analysis of images of vertical ex vivo dermoscopy, the description of visible structures was completed.

### 2.1. Histopathological Classification

In the mixed forms of basal cell carcinomas (BCCs), the superiority rule was applied according to the most unfavorable subtype: aggressive > non-aggressive. In the case of the combination of two non-aggressive subtypes, tumor thickness was considered: nodular > superficial. If the superficial BCC was combined with the nodular, it was classified as nodular BCC, accordingly, the superficial group includes purely superficial BCCs, whereas the nodular group includes nodular and its variants. The aggressive group consisted of all types of high-risk BCCs, whether alone or in combination with some type of low aggressiveness-tumors (superficial or nodular). Squamous cell carcinomas (SCC) were categorized into in situ and invasive. Melanomas were categorized into in situ, superficial spreading and nodular type. 

The level of invasion of BCCs is classified into papillary dermis, reticular dermis, and subcutaneous level, whereas for SCCs and melanomas tumor thickness was determined in millimeters. The width for all three tumor types was also determined in millimeters, and was compared with the tumor width measured on horizontal ex vivo dermoscopy.

Among the independent variables, localization, expression of dermoscopic structures on the vertical aspect, and approximate thickness and width of the tumor measured with a millimeter marker embedded in the contact glass of the dermatoscope, were examined. The obtained data were entered into the Excel database, for further statistical processing and analysis.

### 2.2. Statistical Analysis

The commercial software package (version 22.0, SPSS Inc., Chicago, IL, USA) was used for statistical processing of the obtained results. The histopathological finding was the gold standard of the study. In the analysis of the obtained results, methods of descriptive statistics used were: absolute and relative numbers (n, %), and measures of central tendency (arithmetic mean—X and median—Med). Accuracy in relation to the gold standard was assessed using the method according to Obuchowski [15,16]. Measurement of agreement was assessed by Weighted Kappa or Intraclass Correlation Coefficient (ICC).

## 3. Results

### 3.1. Patients and Tumors Characteristics

The research sample consisted of 53 malignant lesions, obtained from 50 patients with male predominance M = 32 (64%), F = 18 (36%). The age of the included patients ranged from 41 to 93 years (mean age 71.1, median 72). The majority of the patients had one lesion (n = 48, 96%) while the remaining two patients had multiple lesions (one had two, and one patient had three lesions).

The diameter of the included lesions ranged from 2 to 50 mm (mean 12.5 mm, median 10 mm). Most BCCs were of medium thickness (71.9%, 23/32) while superficial (15.6%, 5/32) and thicker (12.5, 4/32) BCCs were less common. The thickness of invasive SCCs ranged from 1 to 5 cm (mean 3.5 cm, median 4 cm), while the thickness of invasive melanomas ranged from 3 to 6 cm (mean 4.43, median 4.35).

Most lesions were located on the head and neck (n = 35, 66%) followed by the trunk (n = 11, 21%), and the extremities (n = 7, 13%).

The final histopathologic diagnosis revealed 32/53 (60.4%) BCCs as follows: low-risk BCCs 26 (superficial 5, nodular 7, nodular-superficial 4, nodular-adenoid 3, nodular-cystic 1, adenoid 3, adenoid-cystic 2, adenoid-superficial 1); high-risk BCCs 6 (infiltrative 3, infiltrative-nodular 2, micronodular-nodular 1). The group of SCCs (15/53, 28.3%) consisted of 13 invasive SCCs, and two in situ (Bowen disease). Two melanomas each, in situ, superficial spreading, and nodular are included in the melanoma group 6/53 (11.3%). 

### 3.2. Basal Cell Carcinomas

**Figure 1:**
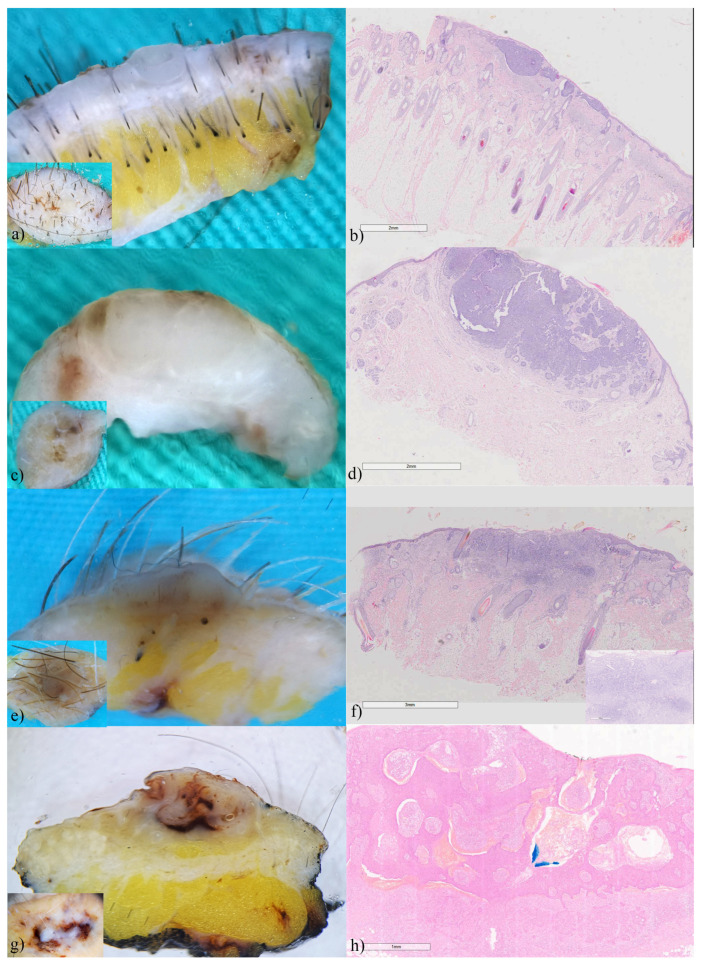
Basal cell carcinomas (**a**) superficial, (**c**) nodular, (**e**) adenoid-superficial, (**g**) nodulo-cystic. ex vivo dermoscopy and histopathology. Horizontal plane, insets: non-specific dermoscopic finding; (**a**) vertical plane: a clearly visible tumor bud invading the papillary dermis; (**b**) Histopathology HEx20: multifocal tumor nests of basaloid cells originating from the epidermis. (**c**) vertical plane: two whitish, deeper tumor islands confluent in the central part; (**d**) Histopathology HEx15: tumor islands start from the epidermis and invade the dermis. (**e**) vertical plane: translucent tumor tissue that reaches the hypodermis; (**f**) Histopathology HEx7, inset ×40: reticulated pseudoglandular structures of basaloid cells. (**g**) vertical plane: raised whitish tumor island, interspersed with hemorrhage in the central part; (**h**) Histopathology HEx20: BCC with hemorrhage in the intratumoral stroma.

Basal cell carcinomas were the most frequent and had the most diverse presentation on the vertical section. The findings of vertical ex vivo dermoscopy showed greater similarities with the histological findings than with the findings of horizontal ex vivo dermoscopy. Therefore, in addition to the horizontal view (Figure 1 insets), the vertical plane of ex vivo dermoscopy, provided additional information that may indicate the BCC subtype. In the superficial variant of BCC vertical ex vivo dermoscopy, and histopathology visualizes tumor buds extending from the basal membrane zone (Figure 1a,b). The existence of tumor islands located deeper in the dermis indicates a nodular BCC or its variants, due to almost identical presentation on histopathology (Figure 1c,d). In adenoid variant, tumor islands showed translucency, without a clear border, because of the reticular distribution of the tumor (Figure 1e,f). In the cystic variant of BCC vertical ex vivo dermoscopy enabled the visualization of the entire tumor island, as well as the contents inside it, and not only its peak (Figure 1g,h). 

**Figure 2:**
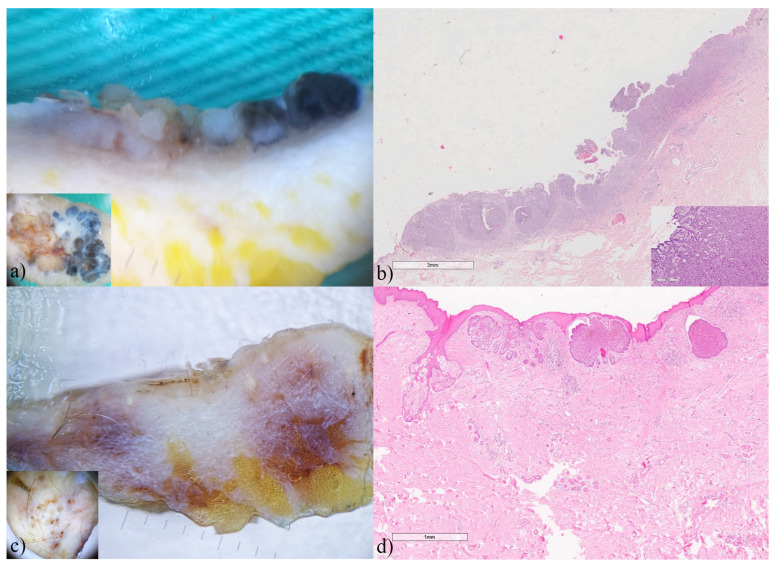
Basal cell carcinomas (**a**) nodulo-adenoid, (**c**) superficial, ex vivo dermoscopy and histopathology. (**a**) horizontal plane, inset: on horizontal ex vivo dermoscopy, we clearly see blue-gray ovoid nests, a few arborizing blood vessels, as well as whitish areas and lines; (**a**) vertical plane: on a vertical section, we could see the exact size, shape, pigmentation, and configuration of the tumor islands; (**b**) Histopathology HEx7, inset ×100: solid tumor islands with pseudoglandular formationes in the dermis. (**c**) horizontal plane, inset: this aspect allows us to see multiple brown dots and numerous whitish areas; (**c**) vertical plane: a thin pigmented line could be seen on the surface of the tumor bud; (**d**) Histopathology HEx20: superficial BCC with slight pigmentation in multifocal tumor nests.

**Figure 3:**
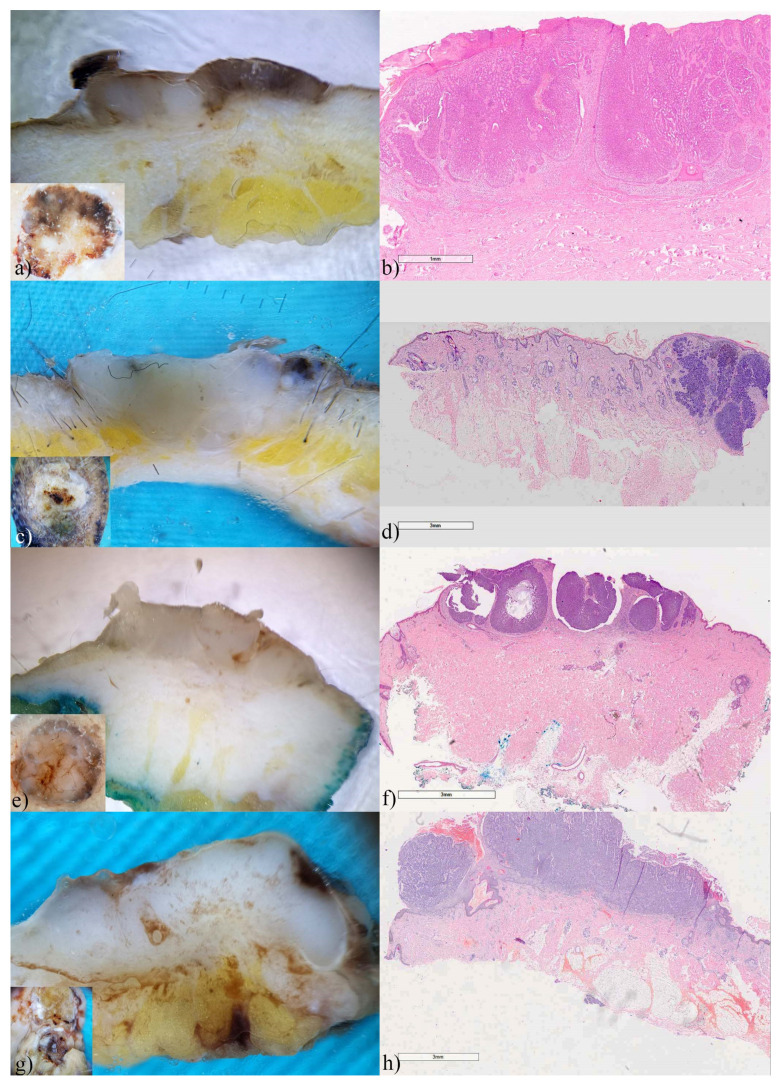
Basal cell carcinomas (**a**) nodular, (**c**) adenoid-cystic, (**e**) nodular, (**g**) nodular-adenoid, ex vivo dermoscopy and histopathology. (**a**) horizontal plane, inset: intense brown pigmentation dominates the findings; (**a**) vertical plane: homogeneously pigmented and whitish tumor island that invades the dermis; (**b**) Histopathology HEx20: focally eroded BCC nodules. (**c**) horizontal plane, inset: central dominance of whitish area with peripheral indigo blue pigmentation; (**c**) vertical plane: translucent whitish tumor island that invades the subcutaneous fat tissue; (**d**) Histopathology HEx7: reticulated pseudoglandular structures of basaloid cells with central cystic formation. (**e**) horizontal plane, inset: translucent, pale pigmented homogenous area as a dominant feature, with a few linear blood vessels; (**e**) vertical plane: a whitish, thick, linear-shaped tumor island that invades the dermis; (**f**) Histopathology HEx10: BCC nodules in superficial part of dermis. (**g**) horizontal plane, inset: non-specific dermoscopic finding with pronounced whitish areas. (**g**) vertical plane: homogenous, thick whitish tumor island, irregularly linear in shape that invades the dermis; (**h**) Histopathology HEx7: solid tumor islands with pseudoglandular formations.

Applying horizontal ex vivo dermoscopy, we encountered two scenarios: cases where the diagnosis was clear (Figure 2 insets), and cases with an inconclusive diagnosis (Figure 3 insets). In cases with a clear diagnosis, we wanted to determine whether the additional application of vertical ex vivo dermoscopy could provide any significant additional information. As we can see in Figure 2, the tumor islands, their shape, color, size and arrangement are clearly visible, both on the vertical dermoscopic aspect and on histopathology, especially in the nodular and pigmented variant of BCC, which allows us to better evaluate the thickness of the tumor as well as the way of spreading of the tumor islands.

In cases with an unclear diagnosis, vertical ex vivo dermoscopy did not contribute to a more precise diagnosis, but as in previous cases, it provided a better insight into the depth of penetration and tumor margins, as well as an excellent correlation (except for colors) with histopathological findings (Figure 3).

**Figure 4:**
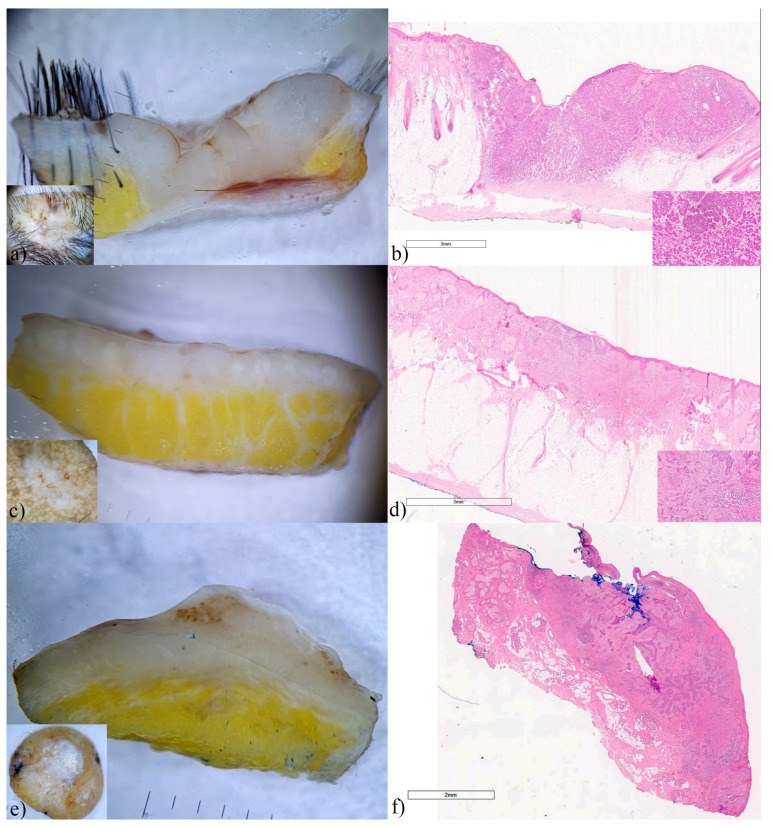
Basal cell carcinomas (**a**) micronodular, (**c**,**e**) infiltrative, ex vivo dermoscopy and histopathology. Horizontal plane, insets: dominance of whitish areas. (**a**) vertical plane: clearly limited tumor tissue that invades the hypoderm; (**b**) Histopathology HEx10: Micronodular BCC, with small tumor nests (inset ×100) invading deeper central parts of the subcutaneous fat tissue. (**c**,**e**) vertical plane: (**c**) flattened and (**e**) raised whitish tumor island that invades the entire thickness of the dermis, with unclear boundaries; (**d**) Histopathology HEx10: Infiltrative BCC, with irregular tumor islands (inset ×100), reaching junction of the reticular dermis with the subcutaneous fat. (**f**) Histopathology HEx10: infiltrative BCC invading superficial parts of the subcutaneous fat tissue.

Further analysis included the ability to differentiate aggressive forms of BCCs using vertical ex vivo dermoscopy. Invasion of tumor islands through the entire thickness of the reticular dermis was visible in all high-risk BCCs, regardless of whether they were single-type or mixed variant (Figure 4), while the lower border of the tumor was clearly observed only in micronodular mixed variant BCC (Figure 4a). 

### 3.3. Squamous Cell Carcinoma

**Figure 5:**
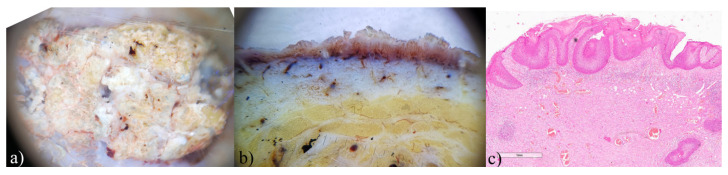
Morbus Bowen, ex vivo dermoscopy and histopathology. (**a**) horizontal plane: dominance of whitish keratin deposits; (**b**) vertical plane: whitish tumor tissue present within the epidermis with numerous polymorphic linear blood vessels; (**c**) Histopathology HEx20: widened epidermis, with full thickness atypia.

**Figure 6:**
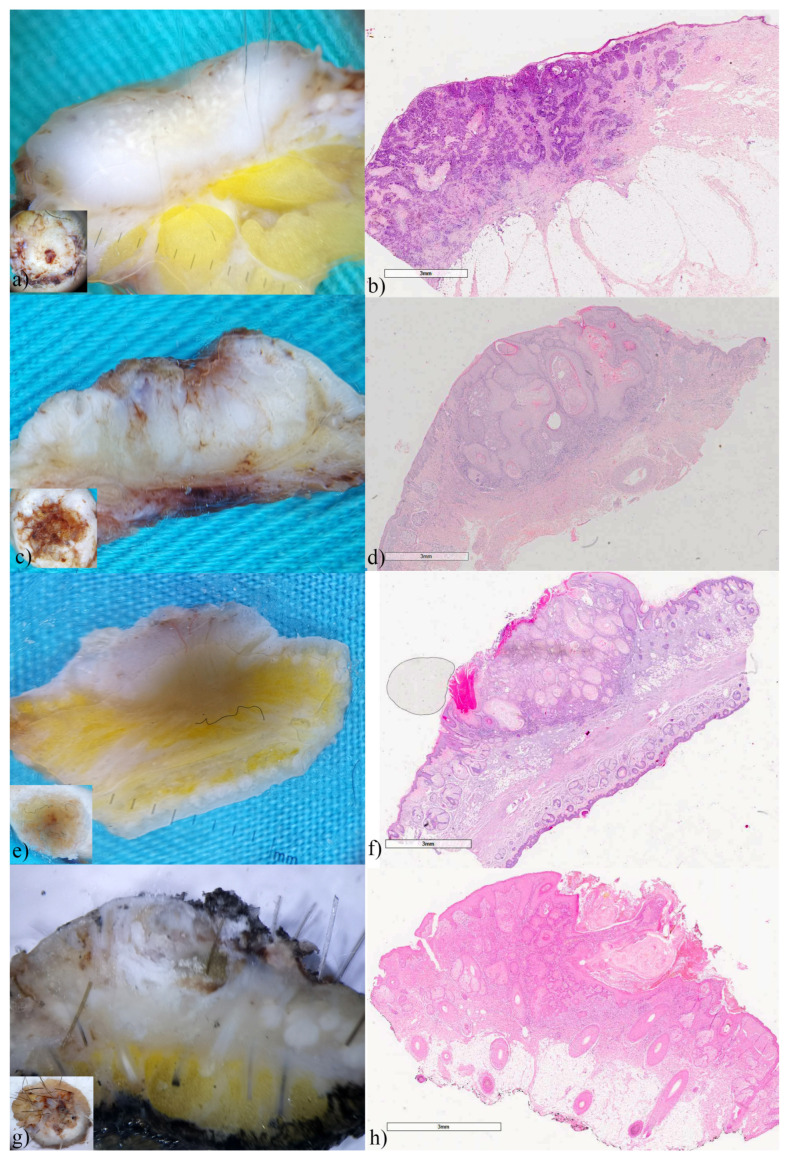
Invasive squamous cell carcinomas, ex vivo dermoscopy and histopathology. (**a**,**c**,**e**,**g**) horizontal plane, insets: eroded whitish and partially pigmented areas. (**a**,**c**,**e**,**g**) vertical plane: similar presentations in the form of thick, whitish tumor nodules with variable levels of penetration. (**b**) Histopathology HEx7: tumor tissue of well-differentiated carcinoma penetrates the reticular dermis into subcutaneous fat. (**d**,**f**) Histopathology HEx7: tumor tissue of well-differentiated carcinoma penetrates the reticular dermis. (**h**) Histopathology HEx10: moderately differentiated SCC (follicular/infundibular type).

Squamous cell carcinoma accounted for almost a third of the analyzed skin malignancies. Of the 15 analyzed SCCs, two were intraepidermal (Bowen’s disease), and 13 were invasive. Horizontal ex vivo dermoscopy was inconclusive in most cases of SCCs (Figure 5a and Figure 6 insets), while vertical ex vivo dermoscopy contributed to a clear visualization of tumor invasiveness, as well as the shape, pigmentation and vascularization of the tumor. 

In Bowen’s disease, by ex vivo dermoscopy, both horizontal and vertical, we clearly see thick whitish tumor tissue, while the vertical section visualizes the involvement of the epidermis, and more intense dermal vascularization (Figure 5). The different presentation of the tumor on the vertical aspect of dermoscopy and histopathological findings should be emphasized. 

In invasive SCCs, on vertical dermoscopy, light pigmentation was uniformly or sporadically present only on the tumor surface, while the whole lesions were represented by bud-shaped or half-moon-shaped, whitish, dense tumor tissue (Figure 6). Whitish streaks and clouds were scattered on the surface or in the deeper layers of the tumor tissue. Blood vessels were rarely present, in the form of a few, scattered irregular lines or converging towards the top of the central part of the tumor (Figure 6e).

### 3.4. Melanoma

**Figure 7:**
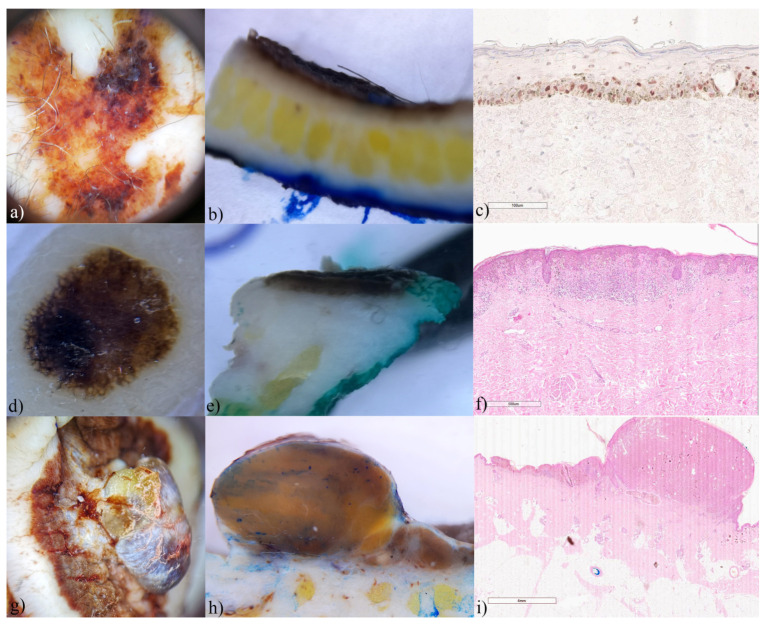
Melanomas, ex vivo dermoscopy and histopathology. (**a**) in situ, horizontal plane: asymmetry of structures and colors; (**b**) vertical plane: thick, superficial pigmented line; (**c**) Histopathology: lentiginous melanoma with PRAME ×200-positive basal and scattered suprabasal melanocytes. (**d**) in situ, horizontal plane: striking radial lines partially present on the tumor periphery; (**e**) vertical plane: thick pigmented line, within the epidermis; (**f**) Histopathology HEx40: shows in situ melanoma and band-like lymphocytic infiltrate in superficial dermis with scattered melanophages. (**g**) superficial spreading melanoma, horizontal plane: characteristic brown pigmentation with whitish fields and vertical growth phase; (**h**) vertical plane: typical melanin color homogeneously present in tumor tissue; (**i**) Histopathology HEx10: showing prominent vertical growth phase and the peripheral radial growth phase.

Melanoma was the least common malignancy in the study. Of the six analyzed melanomas, two were in situ, two were superficial spreading, and the remaining two were of the nodular type. Horizontal ex vivo dermoscopy, despite the loss of vascularization, enabled easy diagnosis of melanoma (Figure 7, insets).

In thin melanomas, the presence of dark brown pigmentation in the form of a continuous line of different lengths and thickness was observed on the vertical section (Figure 7). Also, here as well as with invasive SCCs, histological findings correlated with the findings of vertical dermoscopy. 

**Figure 8:**
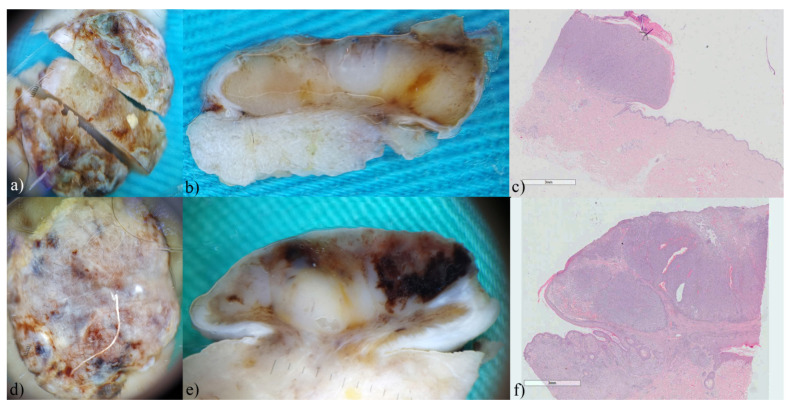
Nodular melanomas, ex vivo dermoscopy and histopathology. Horizontal plane: (**a**) Breslow 3 mm, and (**d**) Breslow 4mm: chaos in colors and structure; (**b**) vertical plane: thick, pale pigmented tumor tissue, linear in shape with whitish central depigmentation; (**c**) Histopathology HEx7: nodular dermal proliferation of atypical melanocytes with ulceration and vascular invasion, without radial growth phase and pigmentation. (**e**) vertical plane: intense melanin pigmentation, non-pigmented islands, and pronounced fibrosis in the form of bright white areas; (**f**) Histopathology HEx7: nodular dermal proliferation of atypical melanocytes with ulceration, without vascular invasion and pigmentation.

In nodular melanomas, horizontal dermoscopy did not indicate the diagnosis as clearly as in thin melanomas (Figure 8a,d). However, on vertical dermoscopy, the recognizable color of melanin in combination with color asymmetry significantly contributed to a more accurate diagnosis (Figure 8b,e). Although horizontal dermoscopy showed chaos in structure and color in all included melanomas, on vertical section only one melanoma showed real chaos in structure and color as we can see on standard dermoscopy (Figure 8e). At the microscopic level, we clearly see monochromatic tumor islands in both nodular melanomas (Figure 8c,f).

### 3.5. Differentiation of Skin Malignancy by Vertical Ex Vivo Dermoscopy

**Figure 9:**
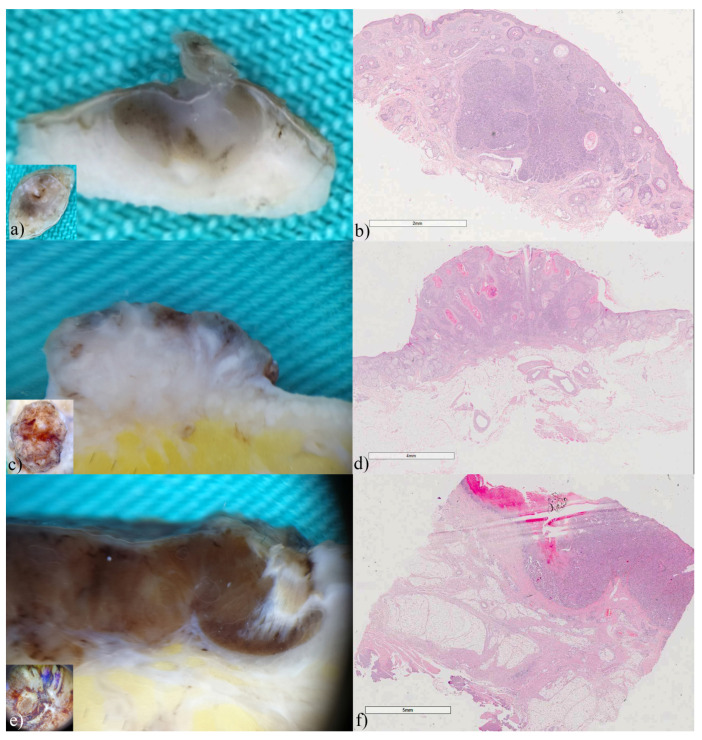
Vertical ex vivo dermoscopy and histopathology, comparative view. (**a**,**c**,**e**) horizontal plane insets: nonspecific dermoscopic presentations (**a**) basal cell carcinoma, adenoid-cystic type: tumor tissue that invades dermis, without thickening of the epidermis; (**b**) Histopathology HEx15: reticulated pseudoglandular structures of basaloid cells with central cystic formation. (**c**) squamous cell carcinoma: tumor that rises with thickening of the epidermis and invasion of the dermis; (**d**) Histopathology HEx7: tumor tissue of well-differentiated carcinoma penetrates the reticular dermis. (**e**) superficial spreading melanoma, Breslow 6mm: a linear-shaped, heavily pigmented tumor that extends to the hypodermis; (**f**) Histopathology HEx7: asymmetric proliferation of atypical melanocytes, with ulceration, focal low-grade pigmentation and vascular invasion.

Further, we analyzed whether vertical dermoscopy may contribute to the distinction of evaluated skin malignancies. As we can see in the comparative view (Figure 9), in BCC we can usually see clearly demarcated tumor island(s) that penetrate deeply, with a relatively thin epidermis (Figure 9a), while in invasive SCCs we found thick, whitish tumors with unclear boundaries, rising in height due to thickened epidermis (Figure 9c). The finding of a typical melanin pigmentation with an asymmetric distribution of whitish structures indicates melanoma (Figure 9e). Vertical dermoscopic correlation with histopathological findings corresponds to specific malignancies (Figure 9b,d,f).

### 3.6. Assessment of Tumor Thickness Using Vertical Ex Vivo Dermoscopy

Statistical analysis disclosed the good values of the thickness accuracy for all three types of malignant tumors evaluated by dermatologists and pathologists. The accurate thickness of BCCs was estimated worse compared to SCCs and melanomas, which had similar values. Both the dermatologist and the pathologist have similar accuracy in relation to the gold standard for the accuracy of BCC thickness, with the dermatologist having higher accuracy and lower standard error. In a direct comparison between the dermatologist and the pathologist, the highest agreement is for the reticular dermis. 

Tumor width was more accurately estimated than thickness in all three types of malignancies. The interobserver agreement was the lowest in BCCs and the highest in melanomas. 

**Figure 10:**
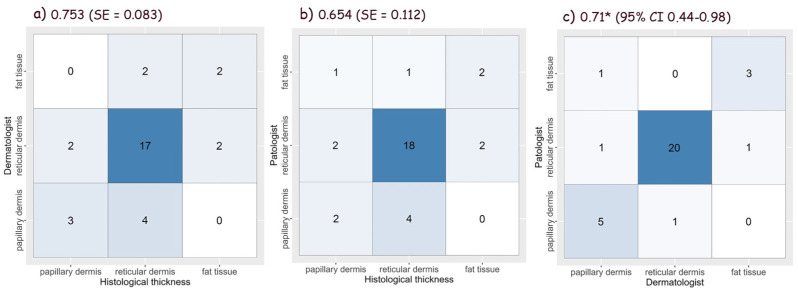
Accuracy of basal cell carcinomas thickness measured by (**a**) dermatologist, and (**b**) pathologist using vertical ex vivo dermoscopy [15,16], and (**c**) interobserver agreement (* Weighted Kappa).

**Figure 11:**
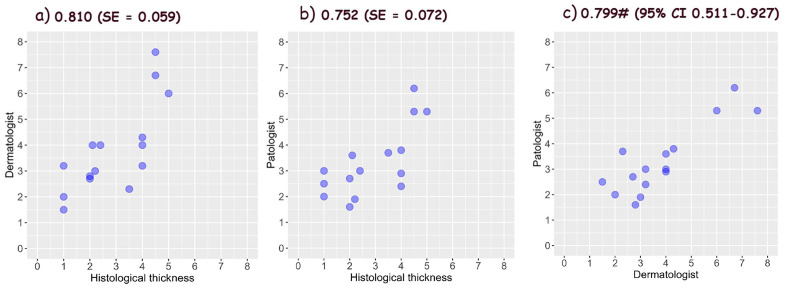
Accuracy of squamous cell carcinomas thickness measured by (**a**) dermatologist, and (**b**) pathologist using vertical ex vivo dermoscopy [15,16], and (**c**) interobserver agreement (^#^ ICC—Intraclass Correlation Coefficient).

**Figure 12:**
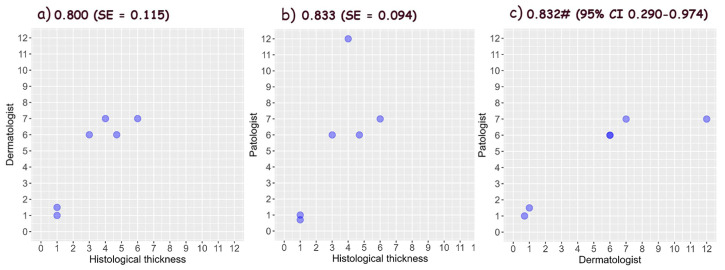
Accuracy of melanoma thickness measured by (**a**) dermatologist, and (**b**) pathologist using vertical ex vivo dermoscopy [15,16], and (**c**) interobserver agreement (^#^ ICC—Intraclass Correlation Coefficient).

The accuracy of the estimated thickness of BCCs, SCCs and melanomas, which were compared with the gold standard by dermatologists and pathologists, and the interobserver agreement are shown in Figure 10, Figure 11 and Figure 12. 

**Figure 13:**
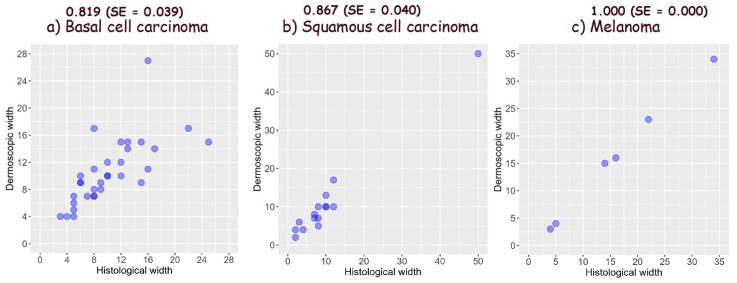
Accuracy of width using vertical ex vivo dermoscopy measured by dermatologist in (**a**) basal cell carcinomas, (**b**) squamous cell carcinomas, and (**c**) melanomas [15,16].

The accuracy of the assessment of the width of the same tumors by dermatologists is shown in Figure 13.

## 4. Discussion

Despite numerous articles confirming the usefulness of dermoscopy during everyday practice, a few articles have been actually published about ex vivo horizontal dermoscopy, while publications about ex vivo dermoscopy in the vertical plane include only sporadical case reports. Published papers on horizontal ex vivo dermoscopy show usefulness for pathologists while for dermatologists and other clinicians dealing with dermoscopy application of horizontal ex vivo dermoscopy does not provide any additional significant diagnostic information compared to standard dermoscopy. Moreover, a certain amount of diagnostic information is lost with the interruption of vascularization at the tissue section, which is the reason for rare or non-usage in everyday clinical practice. Vertical ex vivo dermoscopy has been investigated the least, which is why the main goal of this research was to determine the existence of important additional information that could be significant in the diagnostics of skin malignancy.

### 4.1. Basal Cell Carcinomas

The most diverse clinical presentation, as well as on ex vivo dermoscopy had BCCs, which is associated with a large number of its histopathological subtypes, and a high percentage of its mixed variants. However, regardless of the subtype of BCC and its thickness, on a vertical section, the tumor tissue is mostly seen as a clearly delimited whitish clump/s.

In unclear cases, vertical ex vivo dermoscopy did not contribute to a more precise diagnosis, but as with the clear dermoscopic diagnosis of BCC, it provided a good insight into the descriptive characteristics of the tumor. Regarding the differentiation of BCC subtypes, vertical ex vivo dermoscopy may provide additional useful information. Based on the depth of invasion, we can distinguish superficial from the other BCCs. The central presence of other content (hemorrhage) within the tumor island is more likely to indicate the cystic variant of BCC. The tumor translucency is observed in adenoid variants of BCC, more often pronounced in thinner tumors.

By evaluating BCC on a vertical section, it was also noted that the depth of tumor tissue invasion is positively correlated with a more aggressive subtype, i.e., if the tumor invades adipose tissue, a diagnosis of infiltrative or micronodular BCC is more likely.

Vertical ex vivo dermoscopy of the examined BCCs correlated well with the histopathological findings, except when it comes to colors. Whitish tumor tissue and pigmentation were replaced by standard stains used in routine histopathological tissue preparation.

### 4.2. Squamous Cell Carcinoma

Clinically, all included SCCs had a more uniform presentation, compared to BCC, in the form of keratotic, rarely eroded, partially pigmented nodules/plaques. Ex vivo horizontal dermoscopic presentation, especially of eroded tumors, included various skin malignancies in the differential diagnosis.

In these keratinocyte malignancies, on a vertical section, the tumor tissue is visualized as a half-moon-shaped whitish nodule. The tumor is represented by a thickened, raised epidermis, with unclear borders and initial or deep invasion of the hypodermis. Also, unlike BCC, in SCC whitish structures and blood vessels are often present within the tumor tissue.

The similar presentation of invasive SCC facilitates its recognition and differentiation from other skin malignancies. Furthermore, the vertical aspect of dermoscopy enables the visualization of the depth of invasion, which allows the distinction between non-invasive and invasive forms of SCC.

### 4.3. Melanoma

In melanoma, vertical ex vivo dermoscopy increases the certainty of diagnosis, both in thin and nodular melanomas. On a vertical section, thin melanomas presented as a uniform brown line, while nodular melanomas showed a different shape with recognizable chaos in structure and color.

However, the real importance of vertical ex vivo dermoscopy is in the visualization of the depth of melanoma invasion, which is why its performance should be recommended.

Maia et al. presented a vertical ex vivo dermoscopy of acral melanoma, where tumor nests within intermediate ridges are clearly visible, reaffirming the parallel ridge pattern and the diagnosis of acral melanoma [11]. Our study did not include acral melanoma, so we could not correlate the findings of the same subtype of melanoma.

### 4.4. Assessment of Tumor Thickness Using Vertical Ex Vivo Dermoscopy

An important part of the study was to determine the accuracy of the measured thickness in relation to the histological thickness. Also, we were interested in whether a dermatologist, an experienced dermoscopist looking at a vertical section of tissue with a dermatoscope for the first time, or a pathologist who routinely observes a vertical section of prepared tissue with a microscope, but does not deal with dermoscopy, will provide a better estimate of tumor thickness.

Good assessment of tumor thickness regardless of medical specialty and experience in dermoscopy, as well as good interobserver agreement, encourage the use of vertical ex vivo dermoscopy in obtaining significant data on subclinical tumor spread.

BCCs had the weakest results in the examined parameters, which could be explained by their very different presentation on the vertical aspect of ex vivo dermoscopy. The best agreement was found with medium-thick BCCs, which could be explained by the fact that this thickness was the most common among the evaluated BCCs.

Unlike BCC, SCCs had a similar presentation, which led to a better assessment of tumor depth by researchers. Interobserver agreement was also higher for SCC than for BCC.

Melanoma thickness was best assessed by investigators using vertical ex vivo dermoscopy, and at the same time, interobserver agreement was the highest. The natural brown color of melanin and the whitish structures, easily visible, certainly contributed to the best results in the melanoma group.

The accuracy of tumor width assessed by comparing vertical and horizontal ex vivo dermoscopy was greater in all three types of malignancy compared to thickness. In BCCs, the accuracy of tumor width estimation was inversely correlated with tumor size, i.e., it was better in smaller BCCs, while in SCCs and melanomas, the accuracy of the width estimation was independent of the tumor size. The significance of this finding needs to be further studied.

Currently, several new diagnostic techniques are used, such as videodermoscopy, high-frequency ultrasound, reflection confocal microscopy, and optical coherence tomography. However, these techniques are limited by the depth of penetration, operator experience, availability and diagnostic specificity [17]. Therefore, we would like to emphasize the value of using ex vivo vertical dermoscopy, primarily in the differentiation of skin malignancies.

It should be noted that the resolution of vertical ex vivo dermoscopy is not sufficient for assessing cellular changes or detecting narrow tumor infiltrates.

## 5. Conclusions

In conclusion, vertical ex vivo dermoscopy is fast and inexpensive. It could provide a first and fast orientation about the type of skin tumor and its extent in the tissue. In the future, the technique could be applied not only on formalin-fixed but also on fresh tissue, which will save time, helping surgeons to better estimate the adequacy of resection margins (provisional dermoscopic variant of Mohs technique).

## Data Availability

All data presented can be made available upon request.
